# Interdiffusion and Atomic Mobilities in Rare Earth Alloys: Measurement and Modeling of Dy-Y, Dy-Nd, Sm-Nd, and Sm-Tb Systems

**DOI:** 10.3390/ma18214911

**Published:** 2025-10-27

**Authors:** Wei Yang, Qingzhu Liu, Weiyin Huang, Xiaozhong Huang, Peisheng Wang, Shuhong Liu, Yong Du

**Affiliations:** 1 Powder Metallurgy Research Institute, Central South University, Changsha 410083, China; 2 Hunan Key Laboratory of Advanced Fibers and Composites, Central South University, Changsha 410083, China; 3 Hunan Special Equipment Inspection and Testing Institute, Changsha 410117, China; 4 School of Materials Science and Engineering, Xiangtan University, Xiangtan 411105, China

**Keywords:** rare earth, atomic mobility, interdiffusion coefficient, CALPHAD, CALTPP

## Abstract

Eight diffusion couples were fabricated to systematically investigate the composition-dependent interdiffusion behavior in hcp Dy-Y, Dy-Nd, Sm-Nd, and Sm-Tb binary alloys. The interdiffusion coefficients were determined at two representative temperatures using the Sauer–Freise method based on concentration–distance profiles measured by electron probe microanalysis (EPMA). These experimentally obtained diffusivities, together with available thermodynamic data, were subsequently employed to assess the atomic mobilities of each system by means of the CALTPP (CALculation of Thermo Physical Properties) program within the CALPHAD (CALculation of PHAse Diagrams) framework. The optimized mobility parameters provide a reliable description of the diffusion behavior in all investigated alloys. This reliability is confirmed by the close agreement between the calculated and experimentally measured interdiffusion coefficients, as well as by the strong consistency between the model-predicted and experimental concentration profiles. The present work thus establishes the first set of critically evaluated atomic mobility parameters for these hcp rare-earth binary systems. These results fill an important gap in the kinetic database of rare-earth alloys and lay a robust foundation for future multi-component CALPHAD-based simulations, thereby supporting the design and optimization of advanced rare-earth permanent magnets with improved coercivity and thermal stability.

## 1. Introduction

Rare-earth (RE) permanent magnets are among the most critical functional materials in modern technology, owing to their unparalleled combination of high magnetocrystalline anisotropy, large saturation magnetization, and outstanding energy density. Among them, Nd-Fe-B magnets have become the cornerstone of advanced applications ranging from electric vehicles and wind power generators to high-performance electronic devices, due to their excellent balance between cost, magnetic performance, and processability [1,2,3]. Nevertheless, the widespread utilization of Nd-Fe-B magnets is severely constrained by their relatively low intrinsic coercivity and poor thermal stability [4], which lead to rapid demagnetization under elevated operating temperatures. These limitations restrict their reliability in high-temperature environments such as automotive drive systems and aerospace power devices [5,6].

To overcome these drawbacks, partial substitution of Nd by other rare-earth (RE) elements, particularly heavy rare-earths (HREs) such as Dy and Tb, has been extensively explored as an effective strategy [7,8,9,10,11]. HREs diffuse along grain boundaries [12,13,14] to form HRE-rich shells on the surfaces of Nd_2_Fe_14_B grains [1,15,16]. These shells effectively inhibit reverse magnetic domain expansion, enhance coercivity, and reduce remanence loss [17]. Although HRE diffusion improves coercivity, excessive diffusion leads to the formation of overly thick non-magnetic shells, which can significantly reduce remanence and the maximum energy product [18]. Therefore, precise control over the concentration gradient of diffusing elements is essential to achieving both high coercivity and high remanence [19]. However, studies on interdiffusion coefficients and kinetic databases for rare-earth elements (e.g., Dy-Y, Dy-Nd, Sm-Nd, and Sm-Tb) remain limited.

This study aims to assess the atomic mobility in the hcp phase of the Dy-Y, Dy-Nd, Sm-Nd, and Sm-Tb binary systems. The specific objectives are outlined as follows: (1) to experimentally determine the concentration profiles and interdiffusion coefficients of Dy-Y, Dy-Nd, Sm-Nd, and Sm-Tb diffusion couples at two selected temperatures; (2) to derive and optimize atomic mobility parameters based on these measurements; and (3) to validate the assessed data by comparing calculated and experimental interdiffusion coefficients and concentration profiles.

The insights gained from this work provide fundamental kinetic parameters that are crucial for accurately modeling HREs diffusion processes, thereby enabling the optimization of coercivity and thermal stability in next-generation Nd–Fe–B permanent magnets.

## 2. Experimental Procedure

High-purity rare-earth metals, including yttrium (Y, 99.99 wt.%), neodymium (Nd, 99.9 wt.%), samarium (Sm, 99.99 wt.%), terbium (Tb, 99.99 wt.%), and dysprosium (Dy, 99.99 wt.%) blocks were obtained from Zhong Nuo Advanced Material Technology Co., Ltd., Beijing, China, which were selected as raw materials for the preparation of diffusion couples in this study. The Dy-Y, Dy-Nd, Sm-Nd, and Sm-Tb binary alloys were synthesized using a vacuum arc-melting furnace under a high-purity argon atmosphere. To ensure chemical homogeneity, each alloy button was re-melted at least five times with repeated flipping between melts. The resulting ingots were then sectioned into disks with a diameter of 10 mm and a thickness of 2 mm by wire electrical discharge machining (EDM).

To homogenize the microstructure and minimize the influence of grain size on subsequent diffusion behavior, both the binary alloys and the corresponding pure rare-earth metal blocks were encapsulated in evacuated quartz tubes filled with high-purity argon and annealed at 1073 K for 120 h. The annealed samples were subsequently quenched in cold water and polished to remove surface oxides. For the fabrication of diffusion couples, each binary alloy disk and its corresponding pure metal counterpart were assembled and consolidated by hot pressing in an argon-protected furnace at 873 K under an applied pressure of 300 MPa for 5 s. This process yielded compact diffusion joint cylinders with intimate interfacial contact, and the multi-component diffusion couple fabricated by this method allows multiple diffusion paths to be obtained in a single annealing run, markedly saving experimental time and serving as a high-throughput approach [20,21] for diffusion and phase-equilibria studies.

The hot-pressed cylinders were cut longitudinally along the axial direction to expose the diffusion interface. The sectioned samples were mechanically polished, sealed again in quartz tubes under argon, and subjected to diffusion annealing at the temperatures and durations specified in Table 1. After annealing, the samples were immediately quenched in cold water to preserve the high-temperature diffusion profiles, followed by standard metallographic grinding and polishing.

The concentration distributions across the diffusion zones were characterized using an electron probe microanalyzer (EPMA, JXA-8530, JEOL, Tokyo, Japan). Pure rare-earth reference standards were employed to ensure accurate quantitative calibration. These high-resolution concentration profiles provided the experimental basis for determining the interdiffusion coefficients and assessing the atomic mobility parameters of the studied rare-earth systems.

## 3. Diffusion Model

### 3.1. Determination of Diffusion Coefficients

The interdiffusion coefficients $\tilde{D}\left(c^{*}\right)$ can be quantitatively determined from the experimentally measured concentration profiles using the classical Sauer-Freise method [22] for binary alloy systems, t. This approach is widely employed in diffusion studies, as it allows for the direct extraction of composition-dependent diffusion coefficients without requiring assumptions about constant diffusivity across the diffusion zone. The Sauer–Freise equation is expressed as:

(1) $$\tilde{D}\left(c^{*}\right)=\frac{1}{\left.2t\left(\frac{dc}{dz}\right)\right|z^{*}}\left[\frac{c_{max}-c^{*}}{c_{max}-c_{min}}\int_{-\infty }^{z^{*}}\;\left(c^{*}-c_{min}\right)dz+\frac{c^{*}-c_{min}}{c_{max}-c_{min}}\int_{z^{*}}^{+\infty }\;\left(c_{min}-c^{*}\right)dz\right]$$

where z is the distance along the diffusion direction, $z^{*}$ is the position at which the interdiffusion coefficient is evaluated, t is the diffusion annealing time, $c^{*}$ is the concentration of the diffusing species at position $z^{*}$, $c_{min}$ and $c_{\max}$ are the terminal compositions of the diffusion couple at the two ends.

### 3.2. Atomic Mobilities of Elements

According to the theoretical framework based on the absolute rate theory [23,24], the atomic mobility $M_{B}$ of element B can be expressed in an Arrhenius-type form:

(2) $$M_{B}=\exp(\frac{RTlnM_{B}^{0}}{RT})exp(-\frac{Q_{B}}{RT})\frac{1}{RT}$$

where R is the universal gas constant, $M_{B}^{0}$ the pre-exponential frequency factor, representing the intrinsic attempt frequency for atomic jumps, T the absolute temperature, and $Q_{B}$ is the activation enthalpy for diffusion. The diffusion annealing temperatures (773–1073 K) are well above the magnetic transition temperatures of the pure rare earth elements; hence, magnetic effects on diffusion were not considered.

For disordered solid solution phases, the composition dependence of the frequency factor term $TlnM_{B}^{0}$ and the activation enthalpy $Q_{B}$ can be represented by Redlich–Kister polynomial expansions [25]. The general form is given as:

(3) ΦB=∑i xiQBi+∑i ∑j>i xixj∑r=0mΦBi,jrxi−xjr

where $\Phi_{B}$ denotes either $RTlnM_{B}^{0}$ or the activation enthalpy $Q_{B}$, $x_{i}$ and $x_{j}$ denote the mole fractions of elements i and j, respectively.$\Phi_{B}^{i}$ represents the value of $\Phi_{B}$ for pure element i, ΦBi,jr denotes the binary interaction parameter between elements i and j.

Based on the assessed atomic mobilities, different types of diffusion coefficients can be derived, including trace diffusivity, intrinsic diffusivity, and interdiffusion (chemical) diffusivity. For a mono-vacancy diffusion mechanism and under the assumption that correlation effects can be neglected, the trace diffusivity of element B, denoted as $D_{B}^{*}$ is related to its atomic mobility $M_{B}$ through the Einstein relation:

(4) $$D_{B}^{*}=RTM_{B}$$

where R is the universal gas constant and T is the absolute temperature. This relation highlights the direct proportionality between the atomic mobility and the tracer diffusivity, establishing the fundamental link between kinetic parameters derived from absolute rate theory and measurable diffusion coefficients.

Furthermore, intrinsic diffusivity and interdiffusion coefficients can be expressed as functions of tracer diffusivities and thermodynamic factors. In particular, the chemical diffusivity $\tilde{D}$ for a binary alloy can be expressed as [26]:

(5) $$\tilde{D}=\left(x_{A}D_{B}^{I}+x_{B}D_{A}^{I}\right)=\left(x_{A}D_{B}^{*}+x_{B}D_{A}^{*}\right)\phi$$

where $D_{A}^{I}$ and $D_{B}^{I}$ are the intrinsic diffusion coefficients, $D_{A}^{*}$ and $D_{B}^{*}$ are their respective tracer diffusivities of elements A and B, $x_{A}$ and $x_{B}$ are the mole fractions of elements A and B, ϕ is the thermodynamic factor. The thermodynamic factor ϕ in this work is set to 1 since no binary interaction parameters were assessed in the binary sub-systems.

### 3.3. Self and Impurity Diffusivities

Due to the inherent experimental challenges associated with high reactivity, strong oxidation tendency, and low vapor pressures of rare-earth metals, reliable data on their self-diffusion and impurity diffusion coefficients remain very limited. This scarcity of experimental information poses significant obstacles to the construction of comprehensive kinetic databases. To overcome this limitation, various computational and modeling approaches have been proposed as valuable alternatives for estimating diffusion behavior. Among these approaches, semi-empirical models based on the Arrhenius-type temperature dependence are most widely adopted [26], The general expression is given by:

(6) $$D=D_{0}exp\left(\frac{-Q}{kT}\right)$$

where $D_{0}$ represents the pre-exponential factor associated with diffusion entropy, Q is the activation energy for diffusion. k is the Boltzmann constant, T is the absolute temperature.

To estimate impurity diffusion mobilities, empirical relationships have been proposed that decouple the entropic and enthalpic contributions to diffusion processes. These relationships can be summarized as [26,27]:

(7) $$D_{A}^{A}\times D_{B}^{B}=D_{A}^{B}\times D_{B}^{A}$$

(8) $$Q_{A}^{A}+Q_{B}^{B}=Q_{A}^{B}+Q_{B}^{A}$$

(9) $$ln\left(D_{A}^{0A}\times D_{B}^{0B}\right)=ln\left(D_{A}^{0B}\times D_{B}^{0A}\right)$$

where $D_{A}^{A}$ and $D_{B}^{B}$ are the self-diffusion coefficients of elements A and B. $D_{A}^{B}$ and $D_{B}^{A}$ denote the impurity diffusion coefficients of element A in B, and B in A. $D_{A}^{0A}$, $D_{B}^{0B}$ and $D_{A}^{0B}$, $D_{B}^{0A}$ refer to the pre-exponential factors corresponding to self-diffusion and impurity-diffusion.

In this work, the following strategy was adopted to determine the diffusion parameters for hcp rare-earth elements: (1) Impurity diffusivity approximation: The impurity diffusivity of element B in element A was assumed to be equal to the self-diffusivity of element B. The corresponding self-diffusion parameters of hcp Dy, Y, Nd, Sm, and Tb were obtained from first-principles calculations reported in the literature [28]. This approximation has been widely employed in diffusion studies, as it provides a reasonable estimation in systems where direct impurity diffusion measurements are not available. (2) Polycrystalline diffusivity averaging: To account for the anisotropic diffusion behavior in hcp structures, the effective diffusivity in polycrystalline materials with randomly oriented grains was derived using a statistical averaging method [29]:

(10) $$D=\frac{2}{3}D_{⊥c}+\frac{1}{3}D_{//c}$$

where $D_{⊥c}$ and $D_{//c}$ represent the diffusivities perpendicular and parallel to the crystallographic c-axis, respectively, as determined from single-crystal experiments. This weighted averaging scheme ensures that the derived diffusion coefficients realistically reflect the behavior of polycrystalline aggregates.

By combining first-principles-based self-diffusion data with statistical averaging of anisotropic diffusivities, this strategy enables the consistent estimation of diffusion parameters in hcp rare-earth systems.

## 4. Results and Discussions

**Figure 1:**
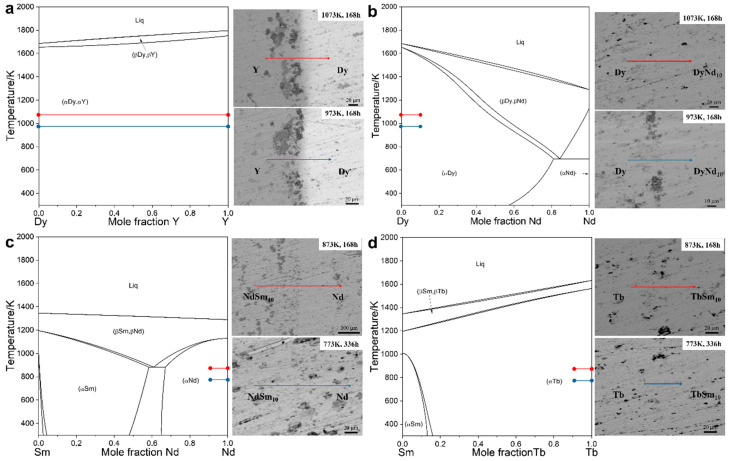
The binary phase diagram, diffusion couples design points, and the BSE image of diffusion couples after annealing.

Figure 1 presents the binary phase diagrams together with the back-scattered electron (BSE) micrographs of the diffusion couples after annealing. The diffusion couples annealed at different temperatures are marked by red and blue horizontal bars, respectively. The line segments in the phase diagrams indicate the nominal composition endpoints of each diffusion couple, while the corresponding bars in the BSE images denote the line-scan regions used for quantitative composition analysis. The elemental concentration–distance profiles perpendicular to the diffusion interfaces were measured by EPMA. To reduce experimental noise and obtain smooth gradients suitable for numerical analysis, the discrete data points were fitted with a Boltzmann-type function, which has been widely demonstrated to describe the sigmoidal nature of diffusion profiles. On this basis, the composition-dependent interdiffusion coefficients were calculated using the Sauer-Freise method [22]. The associated uncertainties were carefully estimated through error propagation analysis [30], ensuring the statistical reliability of the derived diffusion coefficients.

The experimentally determined interdiffusion coefficients were employed to optimize the binary interaction parameters for atomic mobility within the CALTPP program [31,32]. This procedure establishes a consistent link between experimental measurements and the CALPHAD-type kinetic framework. The optimized atomic mobility parameters obtained in this work are summarized in Table 2.

### 4.1. The Dy-Y System

**Figure 2:**
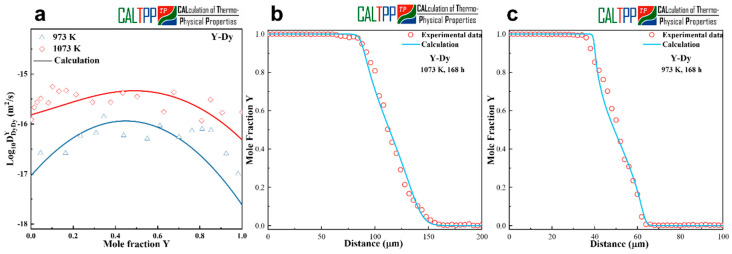
(**a**) Calculated interdiffusion coefficients together with experimental measurements. (**b**,**c**) Concentration profiles for Y-Dy annealed at 973 K and 1073 K for 168 h.

The calculated interdiffusion coefficients for the Dy-Y diffusion couples annealed at 973 K and 1073 K are presented in Figure 2a. As shown, the values obtained from the present atomic mobility assessment are in close agreement with the experimentally derived interdiffusion coefficients, and both exhibit similar compositional dependence. This consistency confirms that the optimized mobility parameters accurately capture the essential diffusion behavior in the Dy-Y binary system.

To further examine the predictive reliability of the assessed mobilities, concentration profiles were simulated using the optimized parameters and compared with the experimental measurements. Figure 2b,c illustrate the comparisons at 973 K and 1073 K after 168 h of annealing, respectively. The model-predicted profiles reproduce the sigmoidal features of the experimentally measured concentration gradients, and the overall agreement across the diffusion zone is satisfactory. This confirms that the presently optimized atomic mobility parameters provide a robust description of diffusion in the Dy-Y system.

It is worth noting that, to the best of our knowledge, no experimental interdiffusion coefficients or atomic mobility parameters for the Dy-Y system—or related rare-earth binary systems—have been reported in the literature. Consequently, the present validation had to rely exclusively on internal consistency checks, namely the comparison between calculated and experimentally measured data from this work. Despite the absence of prior literature for direct benchmarking, the good agreement between model predictions and experimental measurements provides strong evidence supporting the reliability of the assessed mobility parameters.

### 4.2. The Dy-Nd System

**Figure 3:**
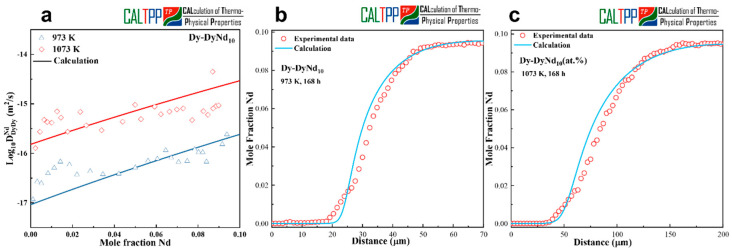
(**a**) Calculated interdiffusion coefficients together with experimental measurements. (**b**,**c**) Concentration profiles for Dy-DyNd_10_ annealed at 973 K and 1073 K for 168 h.

Figure 3a compares the calculated interdiffusion coefficients with the experimentally measured values for the Dy-Nd system at 973 K and 1073 K. The calculated interdiffusivities, derived from the presently optimized atomic mobilities, reproduce the experimental data with satisfactory accuracy across the investigated composition range. This agreement demonstrates that the assessed mobility parameters effectively capture the compositional dependence of diffusion in the Dy-Nd binary system. The optimized atomic mobilities obtained in this work are listed in Table 2.

In addition to diffusivity comparisons, model-predicted concentration profiles were generated for the Dy-DyNd_10_ (at.%) diffusion couples and benchmarked against experimental EPMA measurements. Figure 3a–c show the results after 168 h of annealing at 973 K and 1073 K, respectively. The simulated profiles successfully reproduce the characteristic sigmoidal shape of the experimental concentration distributions and provide good overall agreement throughout the diffusion zone.

Taken together, these results confirm that the optimized kinetic parameters for the Dy-Nd system are both reliable and transferable. The consistency between calculated and experimental data underscores the validity of the present mobility assessment, providing a solid basis for extending the kinetic database of rare-earth binary systems.

### 4.3. The Sm-Nd System

**Figure 4:**
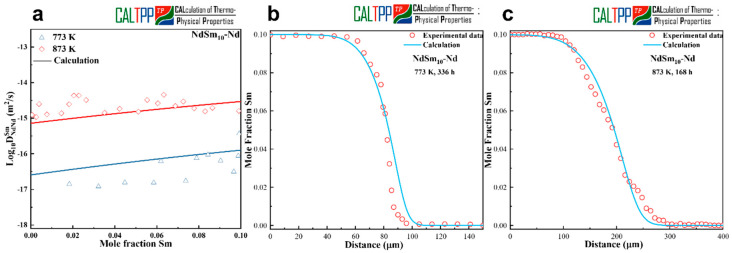
(**a**) Calculated interdiffusion coefficients together with experimental measurements. (**b**,**c**) Concentration profiles for Nd-NdSm_10_ annealed at 773 K for 336 h and 873 K for 168 h.

Figure 4a presents the calculated composition-dependent interdiffusion coefficients in the Sm-Nd system, obtained using the presently optimized atomic mobilities, together with the experimentally determined values. The diffusion couples of Nd-NdSm_10_ (at.%) were annealed at 773 K and 873 K, and the calculated results are shown to be in satisfactory agreement with the experimental data at both temperatures. This consistency demonstrates that the mobility parameters derived in this work are capable of reproducing the essential diffusion behavior of Sm-Nd alloys. The optimized atomic mobilities obtained in this work are listed in Table 2.

To further validate the reliability of the optimized parameters, model-predicted concentration profiles were generated and compared with experimental EPMA results. Figure 4b and Figure 4c display the comparisons at 773 K for 336 h and 873 K for 168 h, respectively. The simulated concentration profiles reproduce the sigmoidal shape of the measured diffusion gradients and agree well with the experimental data across most of the diffusion zone. Nevertheless, slight discrepancies are observed near the Nd-rich end, where the fitting quality is somewhat reduced. This deviation is likely related to surface oxidation effects during annealing, as element Sm possesses a higher affinity for oxygen compared with Nd, leading to localized compositional perturbations at the diffusion couple interface.

Although there is a slight deviation in the terminal composition, the currently evaluated mobility parameters provide a robust description of the diffusion characteristics in the Sm-Nd system. These results further enhance the reliability of the developed rare earth binary alloy kinetic database.

### 4.4. The Sm-Tb System

**Figure 5:**
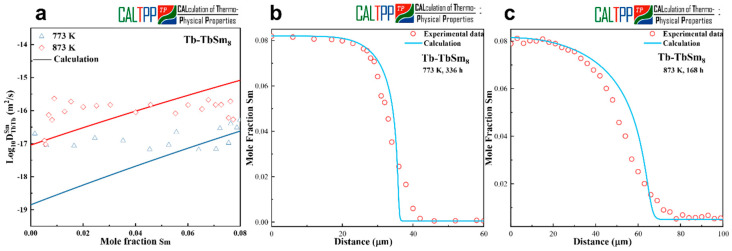
(**a**) Calculated interdiffusion coefficients together with experimental measurements. (**b**,**c**) Concentration profiles for Tb-TbSm_8_ annealed at 773 K for 336 h and 873 K for 168 h.

Figure 5a presents the calculated composition-dependent interdiffusion coefficients for the Sm-Tb system, obtained using the presently optimized atomic mobilities, together with the experimentally measured data for diffusion couples of Tb-TbSm_10_ (at.%) annealed at 773 K and 873 K. At 773 K, the calculated coefficients show good consistency with the experimental results. However, at 873 K, the agreement is less satisfactory, which can be primarily attributed to the high volatility of Sm and Tb at elevated temperatures. Despite efforts to minimize evaporation by sealing the samples in argon-filled quartz tubes, partial material loss during diffusion annealing was unavoidable. Furthermore, due to the tendency of Sm to volatilize during alloy preparation, the nominal TbSm_10_ (at.%) alloy was found by EPMA to actually correspond to TbSm_8_ (at.%), which also contributes to deviations between the simulated and experimental results. The optimized atomic mobilities obtained in this work are listed in Table 2.

To further evaluate the reliability of the assessed parameters, model-predicted concentration profiles were compared with the experimental EPMA measurements. Figure 5b and Figure 5c show the comparisons at 773 K for 336 h and 873 K for 168 h, respectively. The predicted profiles reproduce the general trends of the experimental data, and the optimized mobility parameters can reasonably describe the diffusion behavior of Sm-Tb alloys. Nevertheless, the discrepancies observed at 873 K suggest that the current parameters may not fully capture the true diffusion kinetics under strongly volatile conditions. This highlights the need for improved experimental methodologies to better control composition stability and thereby refine the mobility assessment.

Trace impurities inherently present in rare-earth metals, particularly in heavy rare-earth elements, may exert minor effects on grain-boundary diffusion and local magnetic ordering. Even at trace levels, impurity atoms can segregate at interfaces and form localized impurity magnetic centers, thereby perturbing the surrounding magnetic and diffusion fields [33]. In the present work, the close agreement between the calculated and experimental diffusion profiles indicates that diffusion is primarily governed by the intrinsic mobility of the rare-earth species, and the influence of impurity diffusion can be considered negligible. Nevertheless, the potential role of residual impurities in modifying interfacial or magnetic behavior warrants further investigation in systems with controlled impurity levels.

It is noteworthy that during parameter optimization, temperature-dependent interaction parameters were required for the Dy-Y system, whereas temperature-independent interaction parameters were sufficient to reproduce the diffusion behavior of the Dy-Nd, Sm-Nd, and Sm-Tb systems. This difference implies that diffusion in Dy-Y exhibits stronger temperature sensitivity compared with the other binary systems. Moreover, comparative analysis reveals that the interdiffusion coefficients in the Dy-Nd system are higher than those in the Dy-Y system at both 973 K and 1073 K. Similarly, in the 773–873 K range, the Sm-Nd system exhibits higher interdiffusion coefficients than the Sm-Tb system, indicating that Nd enhances atomic mobility more effectively than Tb in hcp rare-earth alloys.

The atomic mobility data obtained in this work provide a quantitative kinetic basis for optimizing heavy rare-earth diffusion processes in Nd–Fe–B permanent magnets. By integrating the assessed mobilities into CALPHAD-based simulations, the temperature-dependent diffusion behavior of Dy, Tb, Sm, and Y can be predicted with high accuracy, enabling the design of tailored HRE diffusion profiles. An effective coercivity enhancement requires the formation of thin, continuous HRE-rich shells at grain boundaries without excessive diffusion that leads to non-magnetic layer thickening. These findings provide a practical kinetic guideline for controlling diffusion heat treatments and alloy design in next-generation high-coercivity Nd–Fe–B magnets.

## 5. Conclusions

In this study, the interdiffusion coefficients of hcp rare-earth binary systems were systematically determined using bulk diffusion couples and the Sauer–Freise method. Diffusivities were obtained for Y-Dy and Dy-Nd alloys at 973 K and 1073 K, and for Sm-Nd and Sm-Tb alloys at 773 K and 873 K, providing new and reliable kinetic data for these previously unreported systems. Based on the experimental results, the atomic mobility parameters of the hcp Y-Dy, Dy-Nd, Sm-Nd, and Sm-Tb systems were critically optimized using the CALTPP program. The composition dependence of frequency factors and activation enthalpies was described by the Redlich–Kister formalism, yielding internally consistent parameters suitable for CALPHAD-type kinetic databases. Comparisons between the calculated and experimentally measured interdiffusion coefficients, together with the agreement between model-predicted and measured concentration profiles, demonstrate that the optimized atomic mobilities accurately reproduce the diffusion behavior across all four binary systems. The present work, therefore, fills a critical gap in the kinetic data of rare-earth alloys and establishes a robust foundation for CALPHAD-based diffusion modeling. The evaluated atomic mobility parameters can be directly applied to multi-component thermodynamic–kinetic simulations, facilitating the design and optimization of next-generation rare-earth permanent magnets with enhanced coercivity and thermal stability.

## Figures and Tables

**Table 1:** Experimental annealing conditions applied to the diffusion couples.

Diffusion Couples (at.%)	Diffusion Temperature (K)	Diffusion Time (h)
Y-Dy	973	168
	1073	168
Dy-DyNd_10_	973	168
	1073	168
NdSm_10_-Nd	773	336
	873	168
Tb-TbSm_8_	773	336
	873	168

**Table 2:** Summary of the optimized atomic mobility parameters.

Mobility	Parameters (J/mol)	References
Mobility of Y	$\Phi_{Y}^{Y}$ = −260,166.67 − 69.95 × T	[28]
	$\Phi_{Y}^{Dy,Y}$ = 738,973.62 − 655.09 × T	This work
Mobility of Nd	$\Phi_{Nd}^{Nd}$ = −187,533.33 − 75.12 × T	[28]
	$\Phi_{Nd}^{Dy,Nd}$ = 239,840.21	This work
	$\Phi_{Nd}^{Nd,Sm}$ = 78,257.44	This work
Mobility of Tb	$\Phi_{Tb}^{Tb}$ = −233,533.33 − 58.83 × T	[28]
	$\Phi_{Tb}^{Tb,Sm}$ = 240,036.94	This work
Mobility of Dy	$\Phi_{Dy}^{Dy}$ = −243,800.00 − 75.64 × T	[28]
	$\Phi_{Dy}^{Dy,Y}$ = 383,559.15 − 288.13 × T	This work
	$\Phi_{Dy}^{Dy,Nd}$ = 10,166.38	This work
Mobility of Sm	$\Phi_{Sm}^{Sm}$ = −200,333.33 − 81.59 × T	[28]
	$\Phi_{Sm}^{Nd,Sm}$ = 138,422.12	This work
	$\Phi_{Sm}^{Tb,Sm}$ = 438,507.00	This work

## Data Availability

The original contributions presented in this study are included in the article. Further inquiries can be directed to the corresponding author.
